# Echocardiographic markers for left atrial stiffness and left atrial conduction delay in patients with silent paroxysmal atrial fibrillation compared to age-matched controls

**DOI:** 10.1186/s12947-026-00395-1

**Published:** 2026-10-28

**Authors:** Larissa Bastos, Faris Al-Khalili, Magnus Bäck, Aristomenis Manouras, Johan Engdahl, Kambiz Shahgaldi

**Affiliations:** 1 Department of Clinical Sciences, Danderyds Hospital, Karolinska Institutet, Entrévägen 2, Stockholm, SE-182 88 Sweden; 2 Department Clinical Physiology, Danderyd Hospital, Stockholm, SE-182 88 Sweden; 3 Department of Medicine, Karolinska Institutet, Stockholm, SE-171 77 Sweden; 4 Department of Cardiology, Karolinska University Hospital, Stockholm, SE-171 76 Sweden; 5 Department of Cardiology, Danderyd Hospital, Stockholm, SE-182 88 Sweden

**Keywords:** Paroxysmal atrial fibrillation, Left atrial stiffness index, Left atrial activation time, Tissue Doppler, Peak atrial longitudinal strain

## Abstract

**Purpose:**

To study left atrial stiffness index (LASI) and left atrial activation time (LA-AT) changes in an older population with silent paroxysmal atrial fibrillation (PAF) compared to age-matched control.

**Methods:**

Individuals with newly diagnosed PAF and a randomly selected age-matched control group without AF were enrolled from STROKESTOP2-study. All participants had NT-proBNP levels 125–900 ng/L. TTE examinations were analyzed retrospectively offline using dedicated software. Besides conventional echocardiographic measurements, LA-AT was obtained by measuring the time delay between the onset of the P-wave on ECG and tissue Doppler Á peak wave in lateral LA wall. LASI was calculated as the ratio of left ventricular filling (E/é) to peak atrial longitudinal strain (PALS).

**Results:**

Mean age was 76 ± 0.3 years. The PAF group (*n* = 67) presented significant increase of LASI (0.41 (0.29–0.60) vs. 0.32 (0.29–0.43), *P* = 0.001) and prolonged LA-AT (170 (140–180) ms vs. 140 (120–144) ms, *P* < 0.001) compared to controls (*n* = 94). Subgroup analysis in participants with normal LA volume index (LAVI) ≤ 34 ml/m^2^, LASI and LA-AT remained significantly increased/prolonged in PAF (*N* = 27) compared to controls (*N* = 72). NT-proBNP-levels did not differ between PAF (228 (181–348) ng/L) and controls (216 (156–317) ng/L) (*P* = 0.18). ROC curve analysis identified thresholds LASI > 0.54 and LA-AT > 139 msec for discrimination of silent PAF.

**Conclusion:**

Indices reflecting LA stiffness and atrial conduction delay were significantly elevated in recently diagnosed PAF patients. LASI and LA-AT thresholds might non-invasively detect early atrial remodeling without structural enlargement in elderly patients with high risk of PAF and mildly increased NT-proBNP levels.

**Supplementary Information:**

The online version contains supplementary material available at https://doi.org/10.1186/s12947-026-00395-1.

## Introduction

Atrial fibrillation (AF) is associated with structural, electrical and mechanical atrial remodeling leading to impaired atrial function and increased cardiovascular morbidity [1, 2]. Recent research has introduced the concept of atrial failure to describe atrial dysfunction as a result from anatomical, mechanical, and electrical alterations that may impair cardiac performance [2, 3]. In the absence of valvular disease or ventricular abnormalities, atrial pathology changes may induce heart failure (HF) or arrhythmias such as AF [3]. 

Left atrial (LA) stiffness increases with atrial remodeling and is associated with impaired peak atrial longitudinal strain (PALS) [1, 3]. LA stiffness reflects atrial performance in terms of pressure changes needed for a given volume increase which can be calculated invasively [4, 5]. However, transthoracic echocardiography (TTE) offers a non-invasive assessment of left atrial stiffness through the calculation of LA stiffness index (LASI), which has been extensively investigated across diverse HF phenotypes [6–9]. 

Histological and electroanatomic mapping studies have demonstrated increased atrial fibrosis in individuals with AF compared to non-AF, with more advanced fibrosis observed in persistent AF than in PAF [10]. Similarly, LA wall biopsies in patients with AF have demonstrated agreement between histological fibrosis and late-gadolinium enhancement (LGE) with cardiac magnetic resonance imaging (cMRI) [11]. However, TTE provides assessment of atrial electrical alterations by calculating left atrial activation time (LA-AT) [12]. LA-AT has been histologically validated as a marker for atrial remodeling associated with increased atrial fibrosis in patients with post-operative AF [13]. Prolonged LA-AT serves as a significant predictor for incidental and recurrence of AF, particularly observed in advanced age and individuals with history of AF [14–16]. 

Silent AF represent a clinically important entity due to its association with stroke and adverse cardiovascular outcomes [17]. Increasing evidence suggest that structural, functional an electrical atrial abnormality may already be present before the diagnosis for AF [18, 19]. This highlights the need for non-invasive markers capable of identifying early atrial remodeling and improving risk stratification in patients at risk of AF. Recent technological advances in TTE allows non-invasive assessment of LASI and LA-AT. However, limited data exist regarding these parameters in elderly individuals with silent PAF, which may represent an early stage of atrial disease. TTE has the advantage for being non-invasive, more accessible, cost-effective and does not require contrast for LA function assessment. Therefore, we hypothesized to describe early remodeling of atrial failure with new echocardiographic approaches in an elderly general population with silent PAF and increased NT-proBNP levels. Our aim was (i) to evaluate LASI and LA-AT in elderly patients with newly diagnosed silent PAF compared to age-matched control group, and (ii) to investigate whether LASI and/or LA-AT differs in individuals with PAF despite normal LA size, compared to age-matched control group.

## Methods

### Study population

**Fig. 1:**
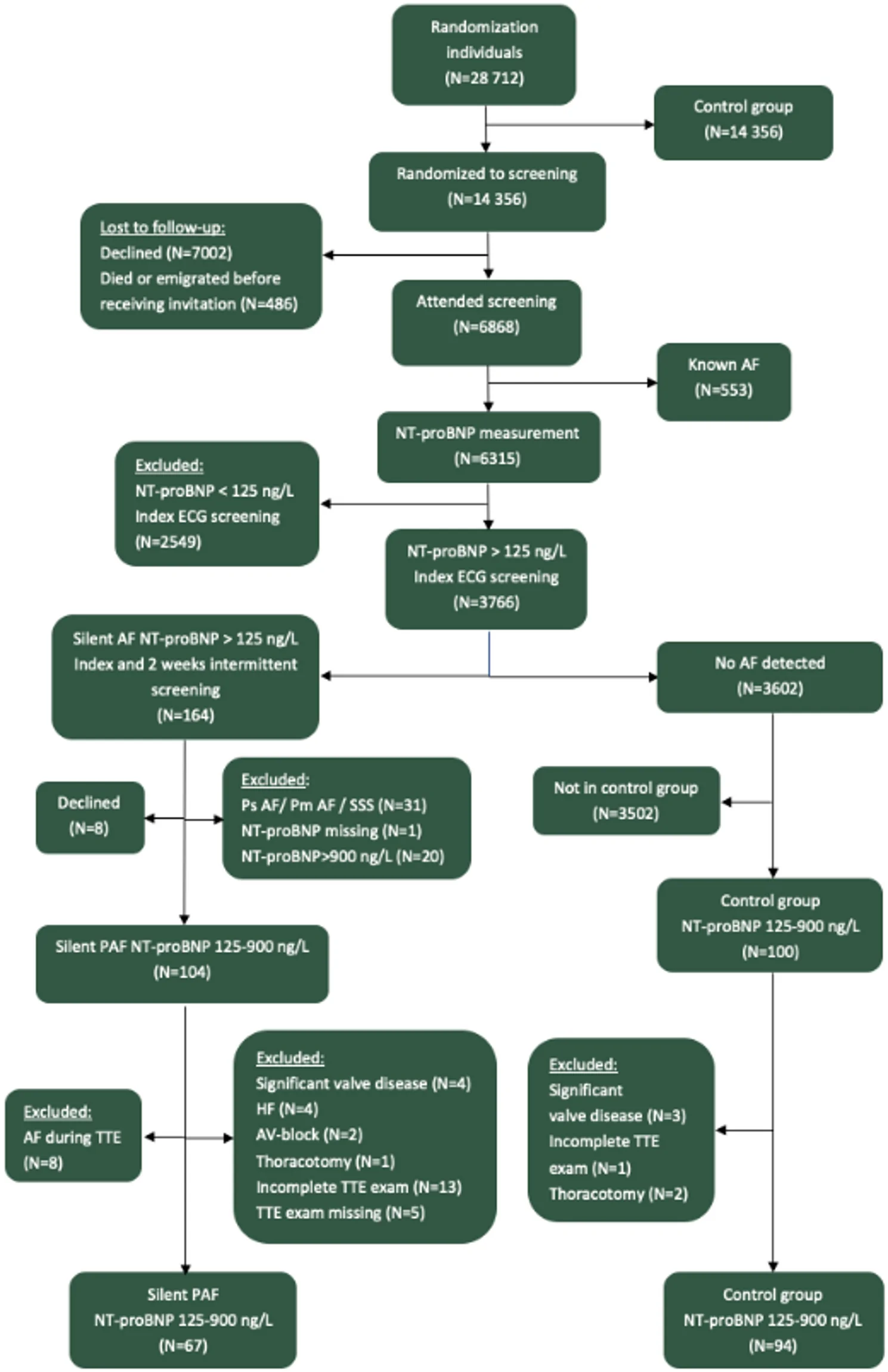
Flowchart for study population. The randomization of individuals derives from STROKESTOP2 - study [20]. (AF; Atrial Fibrillation, PAF; Paroxysmal Atrial Fibrillation, Ps AF; Persistent Atrial Fibrillation, Pm AF; Permanent Atrial Fibrillation, SSS=Sick Sinus Syndrome, HF; Heart Failure, AV-block; Atrioventricular Block, TTE; Transthoracic Echocardiography)

In this retrospective, case-control study, individuals with newly diagnosed silent PAF and a randomly selected control group with no evidence of AF from the STROKESTOP2-study were enrolled [20]. Patients with newly diagnosed PAF and NT-proBNP levels ranging from 125 to 900 ng/L were included. Participants underwent AF screening with handheld-ECG monitoring (Zenicor) during 2 weeks and were asked to record ECG four times/day. Atrial fibrillation was defined as at least one episode of completely irregular rhythm with no organized or regular atrial activity, and a duration of 30 s on one-lead ECG. Patients with ongoing AF during echocardiographic examination or atrioventricular block were excluded. Additional exclusion criteria are reported in the flowchart (Fig. 1). Following exclusion of subjects with previously known heart failure, 100 individuals with NT-proBNP levels between 125 and 900 ng/L and no evidence of atrial fibrillation at screening were randomly selected as controls using the =RAND() function in MS Excel. The inclusion criteria of NT-proBNP levels with 125–900 ng/L defined in STROKESTOP2-study served as an enrichment criterion indicating increased AF incidence risk at NT-proBNP $\:\ge\:$ 125 ng/L. Individuals with NT-proBNP>900 ng/L were excluded from our study due to high cardiovascular risk in the absence of undiagnosed HF and AF [21]. These individuals were prioritized for clinical assessment by a cardiologist according to the STROKESTOP2-study protocol [20]. Information on medical history and comorbidities was obtained from the Swedish National Register. International Classification of Disease 10th revision (ICD-10) was used for the collection of patients comorbidities. NT-proBNP and blood pressure measurements were registered at the inclusion of STROKESTOP2-study, whereas the TTE examinations were performed on a later occasion with median (IQR) 12 [9–16] months apart. However, persistence of PAF diagnosis and absence of documented AF in the control group at the time of TTE examinations was verified through review of medical records, including cardiologist follow-up and ICD-10 codes.

### Conventional echocardiography

The entire cohort underwent TTE examinations (GE Healthcare, Vivid E9, Ms5 transducer) as a part of routine clinical practice according to the study protocol of STROKESTOP2-study between 2016 and 2018 [20]. EchoPAC Software Version 202 was used for offline analysis. Conventional echocardiographic parameters were evaluated during sinus rhythm according to the guidelines from 2015 ASE/EACVI recommendation of chamber quantification and 2016 ASE/EACVI recommendation of diastolic function [22, 23]. All echocardiographic parameters were performed in three consecutive cardiac cycles. A sweep velocity of 100 cm/s was applied for all Doppler images.

### Left atrial stiffness index

**Fig. 2:**
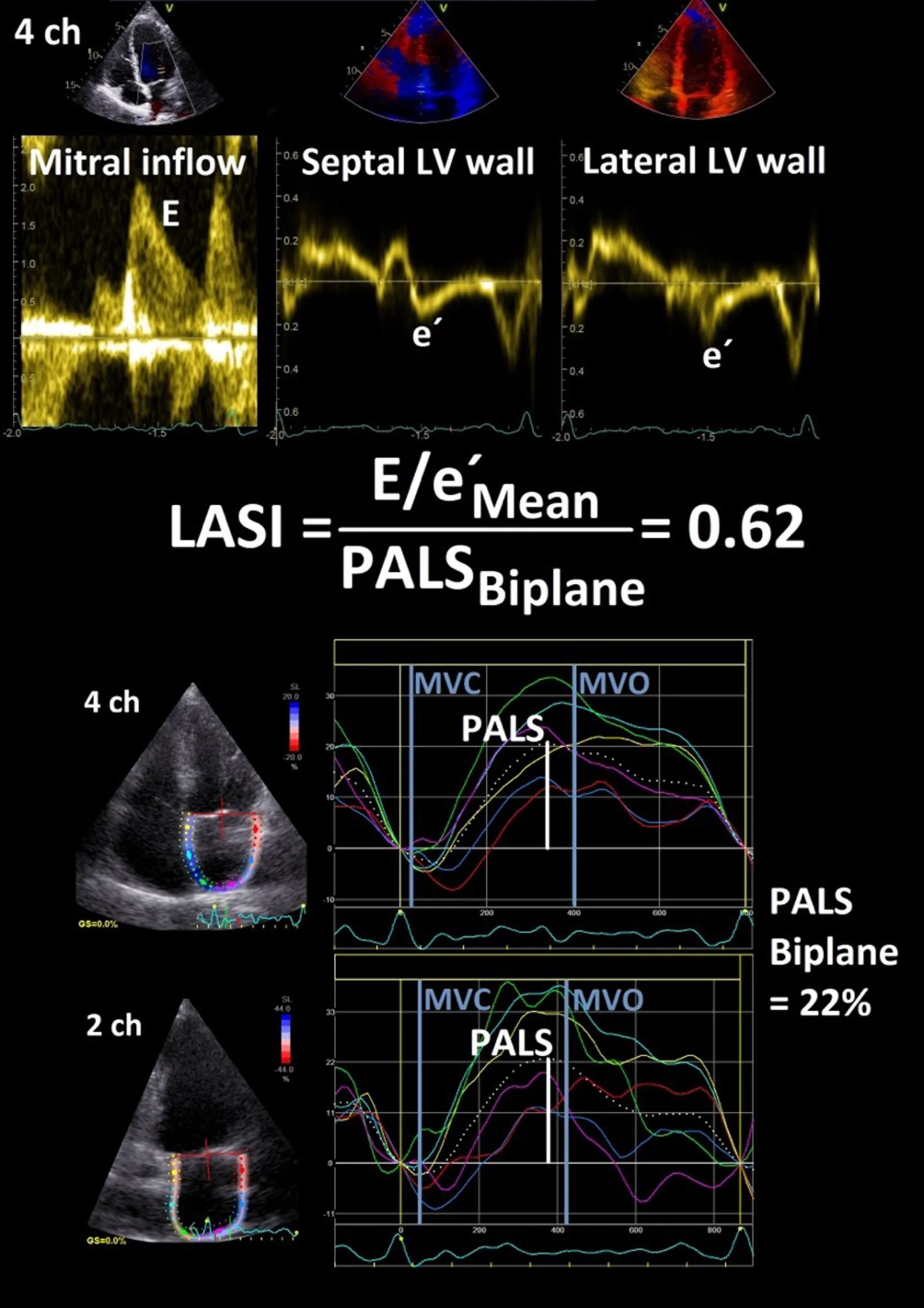
Calculation of left atrial stiffness index (LASI) for an individual with paroxysmal atrial fibrillation, normal left atrial size without diastolic dysfunction. Left ventricular (LV) filling pressures (E/e´_mean_) was calculated with mitral inflow (E) and LV registrations (e´) in septal and lateral wall. PALS was obtained with two-dimensional speckle tracking (2D-ST) biplane. (MVC; Mitral Valve Closure, MVO; Mitral Valve Opening)

Strain analysis was performed using R-R gating, with the zero reference at the QRS-complex. PALS was evaluated in four- and two-chamber view gray scale images with two-dimensional speckle tracking (2D-ST) and quantified as the average of 12 LA segments [24]. PALS was defined as the peak value between mitral valve closure (MVC) and mitral valve opening (MVO) (Fig. 2) [25].

Transmitral E-wave velocity measured as the peak left ventricular (LV) filling during early diastole with pulsed Doppler. LV E/e´ mean ratio was calculated as the ratio of E-wave velocity and the average of the septal and lateral LV e´-wave velocities obtained with tissue Doppler imaging (TDI). Left atrial stiffness index (LASI) was subsequently calculated as the ratio of E/e´ to PALS [4, 5] (Fig. 2).

### Left atrial activation time

**Fig. 3:**
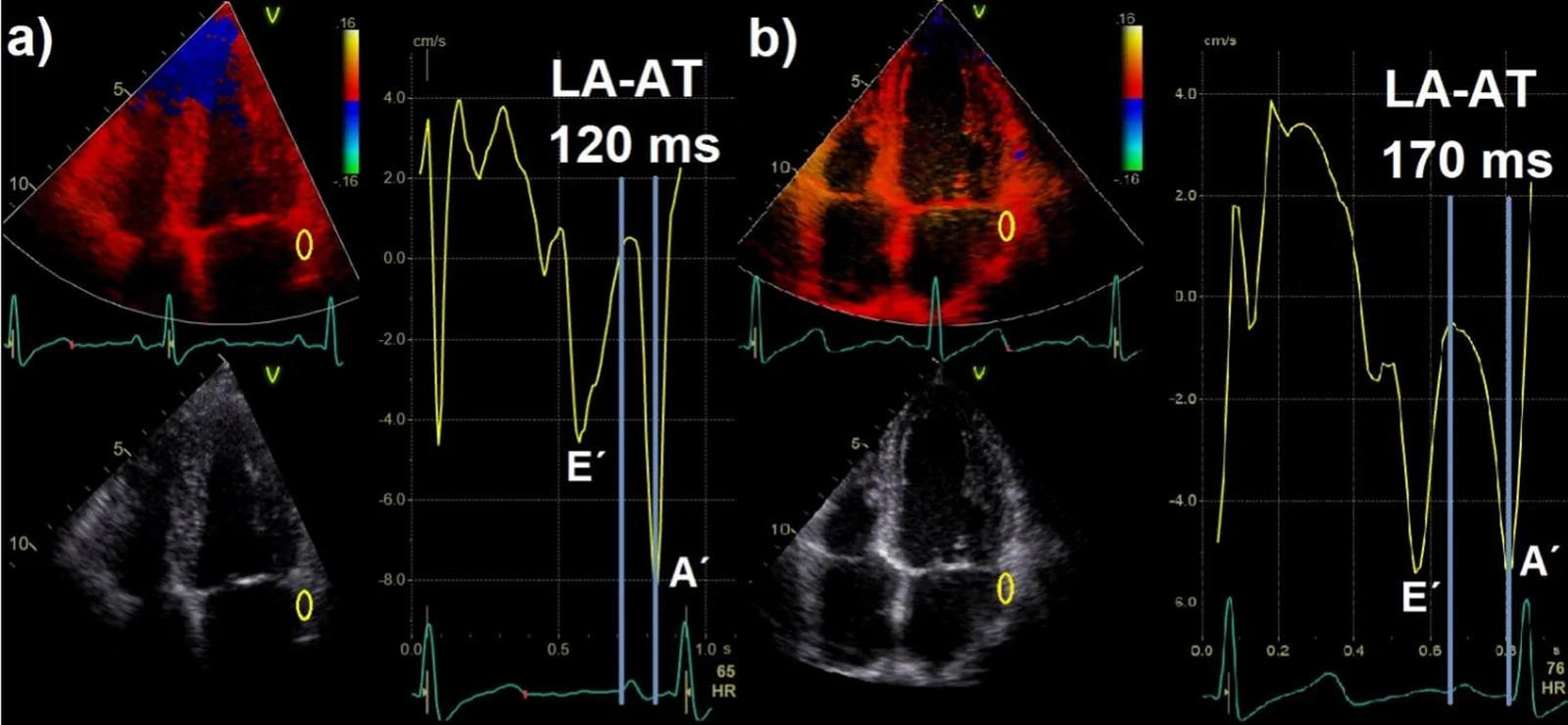
Illustration of left atrial (LA) activation time (AT) in a patient with no history of AF (**a**) compared to an individual with PAF demonstrating prolonged LA-AT (**b**) both normal LA size. (ms; milliseconds)

Left atrial activation time was measured on four- chamber view with color-coded Tissue Doppler Imaging (TDI), collected at the largest extent of three consecutive cardiac cycles. A 10 × 5 mm region of interest (ROI) was placed at the lateral LA wall for recording of tissue velocities during early diastole (E´) and late diastole reflecting atrial contraction (A´). LA-AT was measured as the time interval between P-wave onset using surface ECG to the peak A´ wave [2] (Fig. 3).

### Statistical analysis

All statistical analysis was performed using IBM SPSS Statistics Version 31. Shapiro Wilk´s test defined normal distributed variables expressed as mean with standard deviation (SD). Non-normal distributed data are presented as median with 1st and 3rd interquartile (IQR). Patient demographics and echocardiographic parameters compared between the PAF and control group was investigated with independent Student´s T-test for continuous data with normal distribution. Mann-Whitney U test was applied for non-normal distributed data. Chi-square test and Fishers exact test was applied to analyze differences between proportions, such as variables in patient demographics. Univariate and multivariate linear regression analyses was applied to identify independent factors related to LASI, PALS and LA-AT. Multicollinearity test was evaluated whether independent variables might be correlated to each other affecting dependent variables. Receiver operating characteristics (ROC) curve was performed and the Youden index was applied to define an optimal threshold value for LASI and LA-AT. Spearman´s correlation and Pearson’s correlation was used to test relationship between 2 variables. Kruskal Wallis test adjusted by the Bonferroni correction was applied to investigate if there was a significant difference between LASI and classification of diastolic function (DD). Intra- and inter- variability are presented with Bland-Altman plots and proportional bias was tested with linear regression. Significance value is set as *P* < 0.05. In addition, DeLong for non-parametric statistical method was used to compare the areas under curve for ROC curves from LASI and PALS. Delong test was analyzed with R – statistics version 4.6.0.

## Results

### Analysis of total study population

Out of 164 patients with newly diagnosed AF at screening, 104 patients diagnosed with PAF presenting NT-proBNP levels between 125 and 900 ng/L were included in this study. The final study population comprised 67 individuals with silent PAF and 94 individuals with no evidence of AF, displayed in Fig. 1.

Table 1 presents a homogenous study population in terms of patient demographics, comorbidities, and medication at the time of TTE examination. A significantly proportion of males was observed in the PAF group compared to controls. The PAF group demonstrate larger LV end diastolic diameter (EDD), increased right atrial area index (RAAI) and left atrial volume index (LAVI) compared to the control group. **(**Table 2**)** Controls had normal RAAI while 6% (4/67) of participants in the PAF group showed dilated RA. Although the PAF group displayed significantly increased TAPSE compared to controls, all subjects presented normal right ventricular (RV) systolic function (TAPSE>17 mm). LV global longitudinal strain (LV-GLS), stroke volume index (SVI) and PALS were significantly reduced in the PAF group compared to controls, whereas LA-AT, LASI and PALS were significantly higher in the PAF group. Feasibility measurements were 52% (*N* = 35) for LA-AT and 78% (*N* = 52) for LASI in the PAF group, and 36% (*N* = 34) for LA-AT and 95% (*N* = 89) for LASI in the control group. Feasibility measurements for PALS was 90% (*n* = 60) for the PAF group and 95% (*n* = 89) for the control group.

The increased number of comorbidities in for the entire cohort **(**Table 1**)** including BMI > 30 kg/m^2^, had no relationship with LASI (*r* = 0.17, *P* = 0.05) or LA-AT (*r*=-0.16, *P* = 0.21). NT-proBNP levels had no association with LASI (*r* = 0.16, *P* = 0.05) or LA-AT (*r* = 0.06, *P* = 0.64). LAVI had a moderate relationship with LA-AT (*r* = 0.51, *P* < 0.001) and a weak relationship with LASI (*r* = 0.20, *P* = 0.02). There was no difference in LASI between gender (*r* = 0.19, *P* = 0.03), however a weak relationship was found between LA-AT and gender (*r* = 0.25, *P* = 0.04). PALS had a moderate inverse relationship with LASI (*r*=-0.67, *P* < 0.001) and a weak relationship with LA-AT (*r*=-0.37, *P* = 0.002). In contrast, LASI demonstrated a moderate correlation with E/e´mean (*r* = 0.67, *P* < 0.001). As expected LASI correlated with both E/é and PALS, reflecting its mathematical derivation from these parameters. Therefore, diastolic function was categorized as normal (*n* = 57)/indeterminate (*n* = 9)/dysfunction (*n* = 1) in the PAF group and respectively in the control group (*n* = 56/*n* = 35/*n* = 3) according to the 2016 ASE/EACVI recommendations for diastolic function [23]. Multiple linear regression adjusted for classification of diastolic function and presence of PAF, shows that diastolic classification does not affect LASI between the PAF group and control group. (*P* = 0.64). Binary logistic regression analysis was performed with group status (PAF vs. control) as the dependent variables and gender, LASI, LAVI and PALS as the independent variables. Inclusion of gender in the model altered the odds ratio for LASI by more than 10%, whereas the associations observed for PALS and LAVI remained comparatively stable. Similarly, binary logistic regression analysis was performed with group status (PAF vs. control) as the dependent variable and gender, LA-AT LAVI and PALS as independent variables. Inclusion of gender altered the odds ratio for LA-AT by less than 10%, while no substantial changes were observed for PALS or LAVI.

**Table 1:** Patient demographics of the entire study population, subgroups with normal left atrial volume index (LAVI) and medication at the time of the echocardiographic examination

Patient Demographics	PAF group (*N* = 67)	Control group (*N* = 94)	*P*-value	PAF LAVI ≤ 34 ml/m2 *N* = 27 (40%)	Control group LAVI ≤ 34 ml/m2 *N* = 72 (77%)	*P*-value
Age (Years)	76 ± 0.5	76 ± 0.1	0.28	76 ± 0.4	76 ± 0.1	0.24
Male n (%)	37 (55)	31 (33)	0.005	13 (48)	22 (31)	0.10
NT-proBNP (ng/L) *	228 (181–348)	216 (156–317)	0.18	190 (129–347)	205 (134–287)	0.85
MAP (mmHg) *	99.8 ± 12.2	98.0 ± 10.9	0.33	101 ± 13.5	98.6 ± 11.2	0.41
Heart Rate (beats/min)	67.5 ± 10.4	67.4 ± 10.5	0.96	70.6 ± 10.2	69.3 ± 10.5	0.59
BMI (kg/m^2^)	25.3 (22.3–27.8)	24.3 (22.3–27.8)	0.53	24.9 (20.0–26.3)	24.3 (19.4–28.3)	0.89
Hypertension n (%)	35 (52.2)	46 (48.9)	0.68	10 (37)	31 (43)	0.59
Hyperlipidemia n (%)	8 (11.9)	12 (12.8)	0.88	2 (7.4)	7 (9.7)	1.00
Diabetes n (%)	4 (6.0)	10 (10.6)	0.40	1 (3.7)	7 (9.7)	0.44
Previous Stroke/TIA n (%)	1 (1.5)	7 (7.4)	0.14	0 (0)	3 (4.2)	0.56
CAD n (%)	2 (3.0)	6 (6.4)	0.47	0 (0)	4 (5.6)	0.57
PAD n (%)	1 (1.5)	3 (3.2)	0.64	0 (0)	2 (2.8)	1.00
CKD n (%)	5 (7.5)	2 (2.1)	0.13	1 (3.7)	0 (0)	0.27
COPD n (%)	4 (6.0)	1 (1.1)	0.16	2 (7.4)	1 (1.4)	0.18
OSAS n (%)	2 (3.0)	5 (5.3)	0.70	0 (0)	4 (5.6)	0.21
Anemia n (%)	4 (6.0)	4 (4.3)	0.62	1 (3.7)	2 (2.6)	0.82
**Medicati** **ons**						
BBs n (%)	20 (30)	21 (22)	0.28	5 (19)	13 (18)	1.00
ACEi n (%)	13 (19)	14 (15)	0.45	6 (22)	8 (11)	0.20
ARBs n (%)	19 (28)	19 (20)	0.23	4 (15)	15 (21)	0.50
Statins n (%)	12 (18)	25 (27)	0.20	3 (11)	19 (26)	0.10
CCBs n (%)	15 (22)	23 (25)	0.76	4 (15)	14 (19)	0.77
Diuretics n (%)	9 (13)	12 (13)	1.00	2 (7)	7 (10)	1.00

*Denotes measurements at the time of inclusion of STROKESTOP2 - study. (PAF; Paroxysmal atrial fibrillation, MAP; Mean arterial pressure, BMI; Body Mass Index, TIA; Transient Ischemic Attack, CAD; Coronary Artery Disease, PAD; Peripheral Artery Disease, CKD; Chronic Kidney Disease, COPD; Chronic Obstructive Pulmonary Disease, OSAS; Obstructive Sleep Apnea Syndrome; BBs; Beta Blockers, ACEi; Angiotensin-Converting Enzyme Inhibitors, ARBs; Angiotensin II-receptor Blockers, CCBs; Calcium Channel Blockers)

**Table 2:** Echocardiographic parameters in the study population

Echocardiographic Parameters	PAF group (*N* = 67)	Control group (*N* = 94)	*P*-value
LVEDD (mm)	46.2 ± 4.8	44.5 ± 4.6	0.03
LVMI (g/m^2^)	77.5 ± 16.8	73.6 ± 15.1	0.13
RWT	0.36 (0.33–0.41)	0.37 (0.33–0.41)	0.75
LVEF (%)	59.4 ± 5.6	60.3 ± 5.5	0.32
LV-GLS (%)	19.1 ± 2.5	20.0 ± 2.2	0.04
TAPSE (mm)	25.2 ± 3.7	23.7 ± 4.0	0.02
SVI (ml/m^2^)	39.0 ± 7.1	46.0 ± 8.2	< 0.001
E/A ratio	1.0 ± 0.3	0.9 ± 0.2	0.48
E/e´ mean	9.3 (7.7– 10.5)	9.8 (8.0–11.7)	0.28
PASP (mmHg)	29 (24–34)	29 (25–33)	0.52
RAAI (cm^2^/m^2^)	9.0 ± 1.6	7.8 ± 1.5	< 0.001
LAVI (ml/m^2^)	37.2 ± 9.5	29.0 ± 7.5	< 0.001
PALS (%)	23.6 ± 6.4	30.7 ± 7.6	< 0.001
LASI	0.41 (0.29–0.60)	0.32 (0.25–0.43)	0.001
LA-AT (ms)	170 (140–180)	140 (120–144)	< 0.001

Significance P<0.05. (PAF=Paroxysmal Atrial Fibrillation, LVEDD; Left Ventricular End Diastolic Diameter, LVMI; Left Ventricular Mass Index, RWT; Relative Wall Thickness, LVEF; Left Ventricular Ejection Fraction, LV-GLS; Left Ventricular Global Longitudinal Strain, TAPSE; Tricuspid Annular Plane Systolic Excursion, SVI; Stroke Volume Index, PASP; Pulmonary Artery Systolic Pressure, RAAI; Right Atrial Area Index, LAVI; Left atrial volume Index, PALS; Peak Atrial Longitudinal Strain, LASI; left atrial stiffness index, LA-AT; left atrial activation time)

### Subgroup analysis with normal LA size

Subgroup analysis was performed in all participants with normal LAVI (≤ 34 ml/m^2^). **(**Table 1**) (**Table 3**)** LVEDD and LV-GLS did not significantly differ between the PAF and the control subgroups. Similarly, LV mass index (LVMI), relative wall thickness (RWT), LV Ejection Fraction (EF), E/A ratio, E/e´ ratio mean and PASP did not differ between the PAF group (*N* = 27) and controls (*N* = 72) with normal LAVI for all (*P* > 0.05). However, SVI and PALS were significantly reduced whereas LA-AT and LASI was significantly prolonged/increased in the PAF group compared to the control group. All subjects in subgroup analysis had normal sized RA. Feasibility measurements was 59% (*n* = 16) for LA-AT and 74% (*n* = 20) for LASI in the PAF group, and 39% (*N* = 28) and 94% (*N* = 68) respectively for controls. Based on prior power calculations, a minimum of 23 patients in each group was required. As this was not achieved in the subgroup analysis with normal LAVI, the results may be underpowered and should be considered exploratory. Feasibility for PALS was 89% (*n* = 24) for the PAF group and 94% (*n* = 68) for the control group.

**Table 3:** Echocardiographic parameters in study population with normal left atrial volume index (LAVI)

Echocardiographic Parameters	PAF group LAVI ≤ 34 ml/m^2^ *N* = 27 (40%)	Control group LAVI ≤ 34 ml/m^2^ *N* = 72 (77%)	*P*-value
LVEDD (mm)	44.3 ± 4.9	44.1 ± 4.8	0.88
LVMI (g/m^2^)	71.1 ± 16.3	71.2 ± 14.8	0.98
RWT	0.38 (0.34–0.41)	0.37 (0.32–0.41)	0.30
LVEF (%)	60.5 ± 5.7	60.0 ± 5.6	0.73
LV-GLS (%)	19.1 ± 2.5	20.0 ± 2.2	0.09
TAPSE (mm)	24.9 ± 3.7	23.0 ± 4.0	0.03
SVI (ml/m^2^)	36.4 ± 6.4	44.6 ± 7.0	< 0.001
E/A ratio	0.9 ± 0.3	0.9 ± 0.2	0.80
E/e´ mean	9.1 (7.5–10.5)	9.7 (8.0–11.4)	0.42
PASP (mmHg)	27 (25–33)	28 (25–30)	0.47
RAAI (cm^2^/m^2^)	8.5 ± 1.4	7.6 ± 1.3	0.006
PALS (%)	24.5 ± 8.0	31.1 ± 7.8	< 0.001
LASI	0.42 (0.29–0.56)	0.31 (0.25–0.42)	0.03
LA-AT (ms)	150 (135–170)	138 (120–144)	0.02

Significance P<0.05. (PAF; Paroxysmal Atrial Fibrillation, LVEDD; Left Ventricular End Diastolic Diameter, LVMI; Left Ventricular Mass Index, RWT; Relative Wall Thickness, LVEF; Left Ventricular Ejection Fraction, LV-GLS; Left Ventricular Global Longitudinal Strain, TAPSE; Tricuspid Annular Plane Systolic Excursion, PASP; Pulmonary Artery Systolic Pressure, RAAI; Right Atrial Area Index, PALS; Peak Atrial Longitudinal Strain, LASI; left atrial stiffness index, LA-AT; left atrial activation time)

### ROC curve and regression analysis

Results from univariate and multivariate linear regression analyses for LASI, PALS and LA-AT are presented in Tables 4, 5 and 6 for the entire cohort. NT-proBNP was additionally included in the regression models due to its clinical relevance. TAPSE did not display any significant association with LASI, PALS nor LA-AT (*P* > 0.05).

Multivariate linear regression analysis **(**Table 4**)** revealed that LAVI and diastolic function acted as independent predictor to LASI. As E/e´ and PALS are components of the LASI calculation, they were not included in the multivariate linear regression model to avoid mathematical coupling. Similarly, LV-GLS, LAVI and SVI were independent predictors to PALS **(**Table 5**)**, consistent with our previous findings [26]. For LA-AT **(**Table 6**)**, LAVI was the only independent predictor. No multicollinearity was observed between parameters in multivariate analysis for LA-AT, LASI and PALS, presenting a variance inflation factor (VIF) < 2 across all models.

Receiver operator characteristics (ROC) curve analyses were performed for LASI and LA-AT to evaluate their discriminative performance for silent PAF, adjusted for independent predictors. Derived thresholds for

**Fig. 4:**
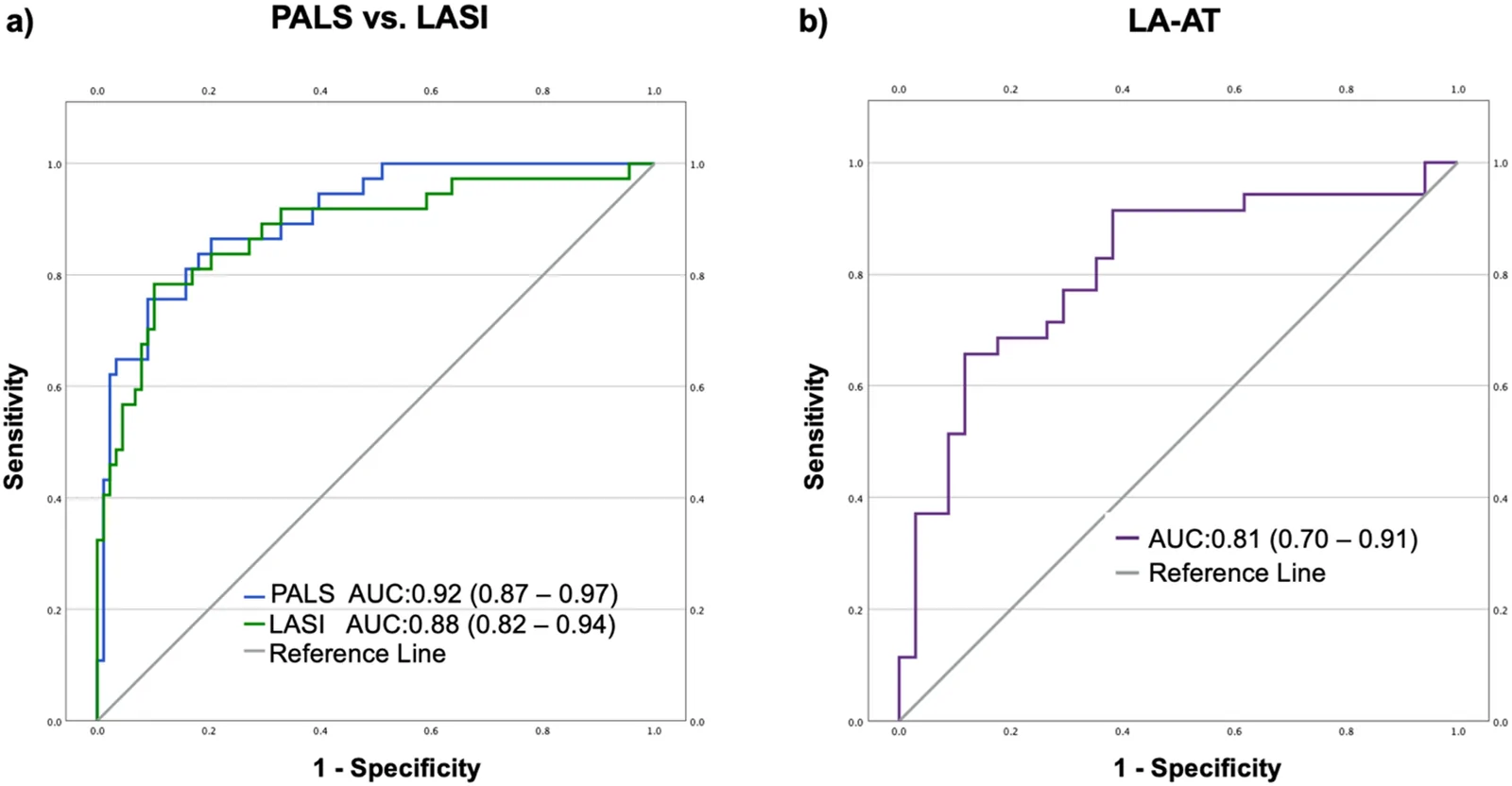
Discriminative performance for detection of silent paroxysmal atrial fibrillation (PAF). (**a**) Receiver operator characteristics (ROC) curve comparison between peak atrial longitudinal strain (PALS) and left atrial stiffness index (LASI). (**b**) ROC curve for left atrial activation time (LA-AT) (b)). Area under curve (AUC) values are presented with 95% confidence intervals

LASI > 0.54 and LA-AT > 139 ms was identified in this cohort as significant discrimination of silent PAF (*P* < 0.0001). LASI adjusted for LAVI and diastolic function had a sensitivity of 75% and specificity 90%, area under curve (AUC) of 0.88 and CI 95% (0.82–0.94) (*P* < 0.0001) **(**Fig. 4a**)**. LA-AT adjusted for LAVI, had a sensitivity of 66% and specificity of 88%, AUC 0.81 and CI 95% (0.70–0.91) (*P* < 0.0001) **(**Fig. 4b**)**.

In addition, ROC curve analysis was performed for PALS adjusted for independent predictors (LV-GLS, SVI, LAVI) to enable comparison with LASI, as PALS is incorporated into the LASI calculation. The findings and clinical significance of PALS in this cohort have been previously reported [26]. In the present cohort, adjusted ROC curve analysis for PALS demonstrated an AUC 0.92 and CI 95% (0.87–0.97) (*P* < 0.0001) for the detection of silent PAF **(**Fig. 4a**)**.

DeLong test was performed to compare AUC-values between two correlated ROC curves (PALS and LASI), demonstrating no significant difference between them (z = 0.79, *P* = 0.43, CI 95% [-0.04 to 0.09]). The comparison of ROC curves is presented in Fig. 4a. Although PALS displays a numerically higher AUC than LASI, this difference was not statistically significant.

**Table 4:** Relationship of left atrial stiffness index (LASI) with TTE parameters and patient demographics in univariate and multivariate linear regression analysis

Study Parameters	Univariate Analysis		Multivariate analysis	
	B-value (95% CI)	*P*	B-value (95% CI)	*P*
Gender	0.04 (-0.02 to 0.10)	0.15	0.043 (-0.015 to 1.02)	0.15
NT-proBNP (ng/L)	< 0.0001 (< 0.0001 to < 0.0001)	0.17	8.192 e^− 5^ (0.0 to 0.0)	0.40
LVEDD (mm)	0.002 (-0.06 to 0.06)	0.96	-	-
LV-GLS (%)	-0.08 (-0.02 to 0.01)	0.23	-	-
SVI (ml/m^2^)	-0.002 (-0.005 to 0.002)	0.29	-	-
LAVI (ml/m^2^)	0.004 (0.000 to 0.007)	0.03	0.003 (0.000 to 0.007)	0.05
RAAI (cm^2^/m^2^)	-0.002 (-0.021 to 0.017)	0.86	-	-
TAPSE (mm)	-0.002 (-0.009 to 0.006)	0.65	-	-
LA-AT (ms)	0.001 (-0.001 to 0.003)	0.20	-	-
Diastolic function	0.105 (0.053 to 0.157)	< 0.001	0.089 (0.035 to 0.142)	0.001

LVEDD; Left Ventricular End Diastolic Diameter, LV-GLS; Left Ventricular Global Longitudinal Strain, SVI; Stroke Volume Index, LAVI; Left Atrial Volume Index, RAAI; Right Atrial Area Index, TAPSE; Tricuspid Annular Plane Systolic Excursion, LA-AT; left atrial activation time

**Table 5:** Relationship of left atrial longitudinal strain (PALS) with TTE parameters and patient demographics in univariate and multivariate linear regression analysis

Study Parameters	Univariate Analysis		Multivariate analysis	
	B-value (95% CI)	*P*	B-value (95% CI)	*P*
Gender	2.40 (-0.20 to 5.00)	0.07	-	-
NT-proBNP (ng/L)	-0.009 (-0.018 to 0.000)	0.04	-0.006 (-0.014 to 0.003)	0.17
Hypertension	-2.62 (-5.17 to -0.08)	0.04	-0.78 (-3.55 to 1.99)	0.60
ACEIs	0.71 (-2.80 to 4.22)	0.70	-	-
ARBs	-3.54 (-6.52 to -0.55)	0.02	-2.52 (-5.68 to 0.65)	0.12
LVEDD (mm)	-3.85 (-6.49 to -1.20)	0.005	-1.91 (-4.80 to 0.98)	0.19
LV-GLS (%)	0.88 (0.33 to 1.44)	0.002	0.61 (0.08 to 1.14)	0.03
SVI (ml/m^2^)	0.27 (0.12 to 0.42)	< 0.001	0.21 (0.06 to 0.37)	0.007
LAVI (ml/m^2^)	-0.29 (-0.42 to -0.15)	< 0.001	-0.21 (-0.35 to -0.06)	0.008
Diastolic Function	-1.72 (-4.18 to 0.74)	0.17	-	-
RAAI (cm^2^/m^2^)	-0.92 (-1.76 to -0.08)	0.03	-	-
TAPSE (mm)	0.25 (-0.08 to 0.58)	0.14	-	-

ACEI; Angiotensin-Converting Enzyme inhibitors, ARBs; Angiotensin II-Receptor Blockers, LVEDD; Left Ventricular End Diastolic Diameter, LV-GLS; Left Ventricular Global Longitudinal Strain, SVI; Stroke Volume Index, SVI; Stroke Volume Index, LAVI; Left Atrial Volume Index, RAAI; Right Atrial Area Index, TAPSE; Tricuspid Annular Plane Systolic Excursion

**Table 6:** Relationship of left atrial activation time (LA-AT) with TTE parameters and patient demographics in univariate and multivariate linear regression analysis

Study Parameters	Univariate Analysis		Multivariate analysis	
	B-value (95% CI)	*P*	B-value (95% CI)	*P*
Gender	-14.0 (-27.3 to -0.63)	0.04	-1.71 (-16.2 to 12.8)	0.82
NT-proBNP (ng/L)	0.009 (-0.04 to 0.06)	0.74	-	-
Hypertension	-7.87 (-21.4 to 5.70)	0.25	-	-
BMI	0.09 (-1.59 to 1.77)	0.92	-	-
LVEDD (mm)	24.9 (11.8 to 38.0)	< 0.001	8.42 (-7.77 to 24.6)	0.30
LV-GLS (%)	-2.20 (-5.40 to 1.00)	0.18	-	
SVI (ml/m^2^)	-0.52 (-1.30 to 0.26)	0.19	-	
LAVI (ml/m^2^)	1.59 (0.94 to 2.23)	< 0.001	1.13 (0.29 to 1.97)	0.009
RAAI (cm^2^/m^2^)	7.23 (3.30 to 11.2)	< 0.001	-	
TAPSE (mm)	1.52 (-0.54 to 3.59)	0.15	-	
PALS (%)	-1.45 (− 2.37 to -0.54)	0.002	− 0.71 (-1.66 to 0.24)	0.14
LASI	26.6 (-14.8 to 68.0)	0.20	-	

BMI; Body Mass Index, LVEDD; Left Ventricular End Diastolic Diameter, LV-GLS; Left Ventricular Global Longitudinal Strain, SVI; Stroke Volume Index, LAVI; Left Atrial Volume Index, RAAI; Right Atrial Area Index, TAPSE; Tricuspid Annular Plane Systolic Excursion, PALS; Peak Atrial Longitudinal Strain, LASI Left Atrial Stiffness Index

### Reproducibility

**Fig. 5:**
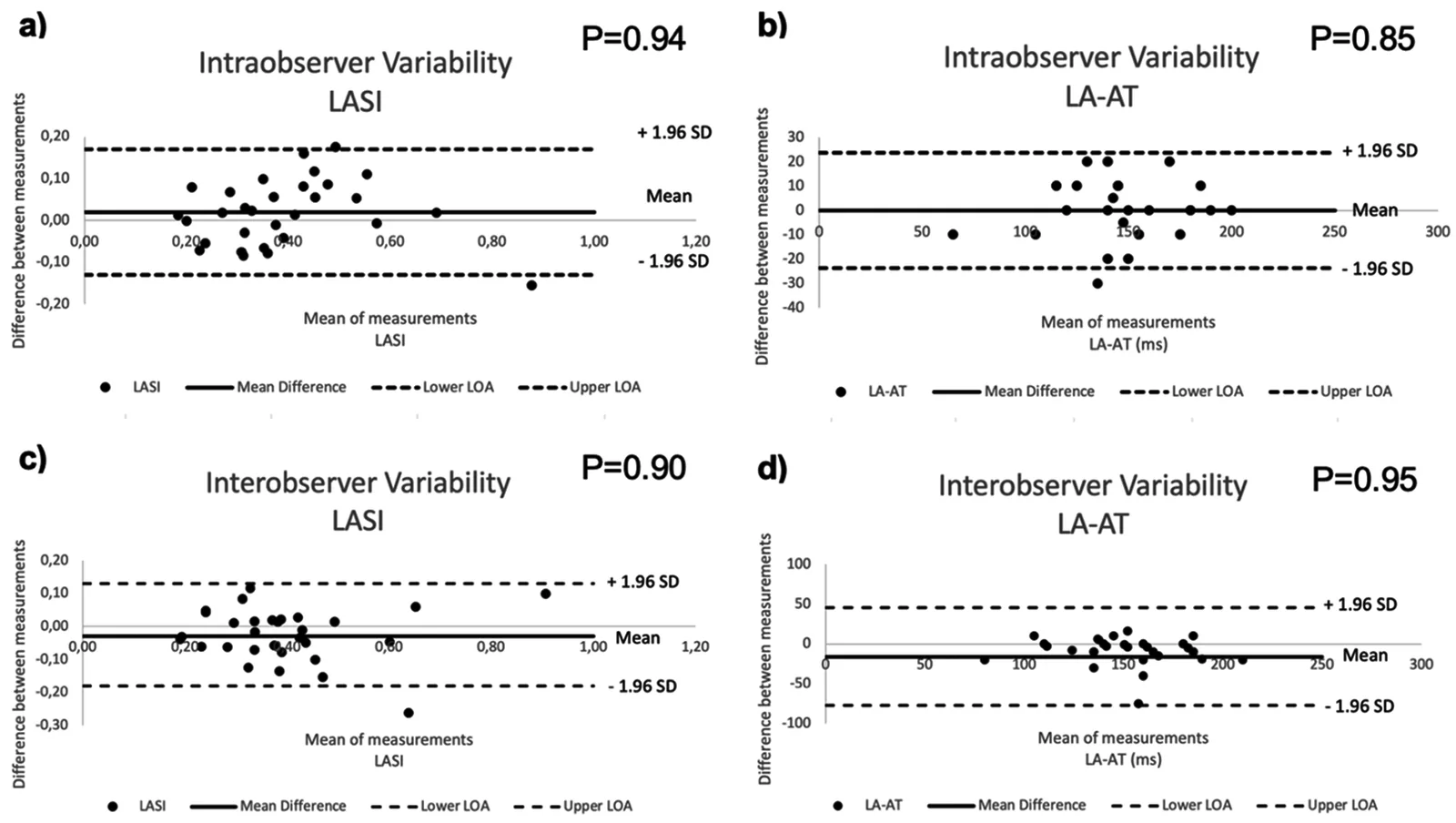
Intraobserver variability for (**a**) left atrial stiffness index (LASI) and (**b**) left atrial activation time (LA-AT). Interobserver variability for (**c**) LASI and (d) LA-AT with corresponding P-values for all

Thirty randomly selected PAF and controls were reassessed for intra- and interobserver variability. Intra-observer variability was measured at least 3 months apart between measurements. Inter-observer variability was performed by a senior cardiac echocardiographer. Bland-Altman plots illustrates an acceptable level of agreement between measurements. **(**Fig. 5) Furthermore, linear regression analysis demonstrates no proportional bias between measurements. Intra-class correlation 0.93 (0.86–0.97) and inter-class correlation 0.92 (0.84–0.96) for LASI and 0.96 (0.91–0.98) respectively 0.89 (0.74–0.95) for LA-AT indicates a god to excellent correlation between measurements. The coefficient of variance (CV) in intraobserver (12%) and interobserver (12%) for LASI indicates good measurement dispersion performance. In contrast a measured intraobserver CV (5%) and interobserver (6%) variability for LA-AT indicates an excellent measurement dispersion performance.

## Discussion

Our study found that echocardiographic markers of left atrial stiffness (LASI) and atrial conduction delay (LA-AT) were associated with silent PAF in an elderly population without overt HF. We demonstrate that in PAF the LASI is increased, and that LA-AT is prolonged even in the absence of enlarged LA. Furthermore, LASI and LA-AT exhibited limited feasibility and high reproducibility with both makers presented significant discriminative ability for silent PAF.

### Echocardiographic differences in the study population

The PAF group displayed larger LVEDD and reduced LV-GLS and SVI despite similar LV EF compared to controls. However, LVEDD, LV-GLS and SVI had no association with neither LASI nor LA-AT. No significant differences were found regarding LV dimensions and LV function between PAF and controls with normal LAVI.

The PAF group in the entire cohort had larger LA and RA size, and reduced PALS as expected compared to the controls group. Thus, we demonstrate that LAVI is as an independent predictor to LA-AT indicating that prolonged LA-AT has an association with increased LAVI in our cohort, resulting in a moderate association. PALS indicating poor LA compliance, did not act as a predictor to LA-AT. Yet, a weak inversely correlation was found in our cohort. Leung M et al. (2018) show a moderate inversely correlation between LA-AT and PALS in their study cohort [27]. 

Furthermore, LAVI and diastolic function classification acted an independent predictor to LASI, presenting that worse diastolic function classification and increased LAVI were independently associated with higher LASI values. Since LASI is strongly correlated with LV filling pressures (E/e´) in our cohort, we ought to investigate the possibility of an association between LASI and diastolic dysfunction. Diastolic function classification did not affect LASI in our cohort which might be explained that the presence of diastolic dysfunction in all participants was low. In addition, LASI had a weak association with LAVI. Our findings are similar to Hatem A.R. et al. (2014) suggesting in a AF cohort that LASI have a moderate relationship with LAVI (*r* = 0.64, *P* < 0.001) [28] However in the present PAF cohort, LASI had a weak correlation with LAVI. The association between NT-proBNP levels and LASI almost reached a statistical significance, nevertheless no association was found between these indices. This might be explained that NT-proBNP levels were predefined at the beginning of the study to minimize the difference between the PAF group and controls.

### Left atrial stiffness indices in PAF

Evidence of increased LASI and reduced PALS in PAF patients has been previously shown in a PAF cohort (*n* = 64) with mean age 57$\:\pm\:$13 years reported by Yoon YE et al. (2012). In the same cohort, LASI demonstrated a moderate correlation with LAVI (*r* = 0.52 *P* < 0.001) [29] Our findings are consistent with these previous observations. While LASI demonstrated simply a weak correlation with LAVI in our cohort, LAVI remained an independent predictor of LASI in multivariate analysis, suggesting that structural atrial remodeling may contribute to LASI. Furthermore, we show that even in the absence of significant anatomical remodeling as reflected by normal LAVI, there is a significant elevation of LASI in PAF as compared to non-AF individuals. In healthy population aged over 60 years of age, the reference value of LASI has been estimated to a median (IQR) of 0.24 (0.18–0.29). Sugimoto et al. (2018) demonstrated an age-related increase in left atrial stiffness index, with LASI reaching 0.55$\:\pm\:$0.09 as the highest expected value of normality. This finding suggests a potential role for LASI as an indicator of subclinical LA dysfunction among healthy individuals aged over 60 years [30]. However, there is limited knowledge regarding whether LASI can serve as a marker of silent PAF in the elderly population with increased NT-proBNP levels. The echocardiographic non-invasive LASI calculation has previously been shown to provide superior prognostic information regarding AF recurrence compared to the invasively derived LASI calculation in AF patients > 60 years old [5]. Importantly, in our cohort, LASI demonstrated a discriminative threshold of > 0.54 for silent PAF, which is close to the upper limit of normality previously reported by Sugimoto et al. (2018) [30] In multivariate analysis, diastolic function classification was independently associated with LASI in our cohort, underscoring the close pathophysiological link between atrial stiffness and left ventricular filling abnormalities. However, this did not explain the higher LASI observed in patients with PAF compared to controls. Adjustment for diastolic dysfunction and the presence of PAF did not attenuate the difference in LASI between groups, indicating that LASI elevation in the PAF group is not solely driven by diastolic dysfunction. This suggests that LASI may reflect additional pathophysiological mechanism beyond diastolic dysfunction in our cohort.

In addition, there was no significant difference in estimated LV filling pressures (E/e´) between the PAF and control groups. Still, it is important to emphasize that LASI represents a composite index reflecting both LV filling dynamics (E/e´) and left atrial strain (PALS), rather than a direct measure of intrinsic left atrial stiffness. Thus, changes in its individual components may contribute to increased LASI values. Given the low prevalence of diastolic dysfunction in our cohort, alterations in LV fillings are unlikely to be the primary driver of the observed LASI differences between groups. Indeed, the presence of left atrial dysfunction reflected by significantly reduced PALS in the PAF group compared with controls, appeared to be the major contributor to the elevated LASI values. The findings on the present study also suggest a potential confounding effect of gender on the association between LASI and PAF status. This may partly reflect the composite nature of LASI through inclusion of PALS together with the lower feasibility of LASI analysis compared with PALS, particularly in the PAF group.

In summary, LASI may be better understood as reflecting atrio-ventricular coupling and left atrial functional impairment, rather than isolated alterations in atrial stiffness. This distinction is important when interpretating the physiological significance of the findings in our study. Our findings suggest subtle left atrial functional impairment, as indicated by LASI, which may reflect early atrial remodeling and contribute to the development of AF. Similar findings were observed in individuals with PAF and normal LAVI, although these results should be interpreted with caution given the limited sample size.

### Left atrial conduction delay in PAF

In our cohort, of elderly with mildly increased NT-proBNP levels, PAF displayed prolonged LA-AT, a marker that has previously been associated with LA fibrosis. Although this association was not directly validated in our study. Notably, prolonged LA-AT was observed even in individuals with normal LAVI, although interpretation is limited by the small sample size. In fact, in our study LA-AT entailed significant discriminative ability for silent PAF. As a marker for atrial conduction delay, LA-AT may reflect underlying left atrial electro-mechanical functional impairment and structural atrial remodeling. Since LAVI is an established marker for LA remodeling, its association with prolonged LA-AT in the present study may further support the concept of progressive atrial remodeling. Our findings are supported in previous research suggesting that individuals with PAF displays prolonged LA-AT, predictive for new-onset of AF [2, 31, 32]. Weijs B. et al. (2011) confirmed that a group (*n* = 205) of older individuals (mean age 67 $\:\pm\:$11 years) with prolonged LA-AT > 157 ms have a higher prevalence of AF, hypertension, CAD, significant valve disease, increased usage of oral anticoagulation, beta-blockers, nitrates, ACE-inhibitors and diuretics. Yet, multivariate linear regression analysis in the same study, revealed that LA-AT was independently prolonged in patients with hypertension, valvular disease, higher age and a higher BMI [16]. In contrast, hypertension and BMI were not independently associated with prolonged LA-AT in our cohort. This discrepancy may be explained by the comparatively healthier study population in the present study, characterized by an age-matched design, absence of significant valvular disease nor presence heart failure and lower burden of cardiovascular comorbidities. Moreover, the association between LA-AT and PAF status in our cohort, remained unchanged after inclusion of gender, suggesting that the observed findings were unlikely to be influenced by sex differences between groups.

In a multicenter study, a threshold presented of LA-AT > 145 ms may identify incidental AF in a cohort with cryptogenic stroke (58$\:\pm\:$12 years) with cardiovascular comorbidities [32] The LA-AT derived threshold for predicting a specific subtype such as silent PAF has not been published earlier and is shortened compared to LA-AT thresholds for AF overall. This might be explained that PAF as the initial AF state, presumably reflecting the beginning of LA conduction delay progression. LA-AT is shown to gradually prolong with AF burden, associated with increased fibrosis as AF progresses from PAF to persistent AF [27, 33]. 

### Clinical impact

Atrial cardiomyopathy has been increasingly recognized in recent research [3, 19, 34, 35]. Within this framework, AF should not only be regarded as an isolated arrhythmia, but rather as a clinical manifestation of progressive underlying atrial disease [35]. Accordingly, a recent ESC consensus statement (2025) describes atrial cardiomyopathy as a gradual transition from healthy atrial structure and function towards advanced atrial failure [19]. The statement further presents atrial markers obtained though multimodal imaging. Within echocardiography, although LAVI remains a well-established marker of atrial remodeling, additional parameters such as PALS and LA-AT are also highlighted as indirect indicators of atrial dysfunction [19]. Atrial cardiomyopathy is characterized by electrical and mechanical atrial dysfunction resulting from underlying pathological changes which may promote atrial enlargement and fibrosis [3]. These abnormalities may progressively develop over time, before clinically manifestation such as AF and heart failure becomes evident [19, 35]. In this context, the present findings of elevated LASI, prolonged LA-AT and reduced PALS may reflect early functional and electro-mechanical atrial remodeling in individuals with PAF, even observed in cases with normal LAVI.

Although no echocardiographic guideline defined cut-off values currently exist for LASI and LA-AT, increasing evidence supports their potential utility in the assessment of atrial remodeling and AF-related dysfunction. For example, Muller et al. (2021) suggested that prolonged LA-AT is associated with increased atrial fibrosis and electrical remodeling, strongly linked to the development and maintenance of AF [2]. LA-AT can also assist in identifying areas of delayed or fragmented activation, which may be targets for ablation. A shorter LA-AT post ablation may indicate successful electrical remodeling [15, 36]. 

LA-AT reflecting atrial mechanical dysfunction was significantly prolonged in AF patients who developed TIA or stroke [37]. Increased LA-AT may also be present in patients with subclinical AF, a potential cause of cryptogenic stroke. Therefore, LA-AT is not only highly reproducible, but also providing additional risk stratification of stroke and may be useful to guide anticoagulation therapy decisions in patients upon first diagnosis of AF [37, 2]. However, LASI might face challenges, before entering the clinical setting as a predictor for incidence of AF in the general older population. LASI is a robust method for the detection of LV diastolic dysfunction since LASI strongly correlates with increased LV end diastolic fillings pressures measured invasively with cardiac catheterization [9]. The general older population has not only a greater probability of AF incidence but also an increased probability of diastolic dysfunction due to either HF and/or valvular disease. Kim et al. (2023) demonstrated that higher LASI in elderly patients were most likely to have AF, hypertension, diabetes, chronic kidney disease and higher NT-proBNP values in a study population with diastolic HF [7]. In the contrary, measuring LASI may be limited as a discriminative factor for silent PAF and might not be applicable in elderly individuals presenting HF, since these patients presumably already present an increased LASI. Therefore, LASI might be useful in patients with high-risk AF in the absence of HF, as indicated by our findings in this study.

Nevertheless, LASI and LA-AT demonstrated high reproducibility in our study, although their feasibility was limited. For instance, LA-AT measurements require high-quality ECG to accurately identify the onset of the P-wave, as well as adequate tissue Doppler imaging of the lateral LA wall. In contrast, LASI calculation is dependent on multiple variables compared to LA-AT and is therefore more complex than LA-AT. This includes reliable measurements of e´ velocities from both septal and lateral LV wall, as well as transmitral E-wave velocity, in addition adequate visualization of the entire LA wall in biplane to ensure accurate PALS measurements. An additional consideration is that LASI is a composite parameter derived from multiple measurements. Thus, errors in any individual component may propagate and influence the overall estimate. These limitations may restrict the routine clinical applicability of LA-AT and LASI. However, it should be noted that the present study was retrospective, and image acquisition was not specially optimized for these parameters. Prospective studies or clinical examinations with predefined imaging protocols may improve feasibility and allow more consistent assessment. From a diagnostic perspective, both LASI and PALS demonstrated high accuracy for detecting silent PAF, with PALS showing numerically higher discriminative performance (AUC 0.92 vs. 0.88), even if the difference between the ROC curves was not statistically significant. This suggest that while LASI captures structural-functional coupling, atrial myocardial deformation parameters such as PALS may be more sensitive in identifying early atrial dysfunction preceding overt arrhythmia. Due to its simpler acquisition and lack of dependence on multiple variables, PALS may offer a more practical and less error-prone parameter for clinical use in comparison to LASI.

However, considering the retrospective case-control design, the ROC-derived thresholds in our study should be considered as exploratory and hypothesis-generating. These thresholds reflect discriminative performance for silent PAF and require external validation. With further validation, these parameters in combination with advanced echocardiographic techniques may contribute to improved risk stratification in clinical practice.

It’s essential that further research is needed supporting the clinical utility of both LASI and LA-AT to serve as indicators of AF. In addition, our study confirms elevated LASI and prolonged LA-AT in elderly population with PAF and mildly increased NT-proBNP values. This pattern was even observed in cases with normal sized LA, suggesting that these indices may reflect LA dysfunction in the early stage of PAF. These findings warrant further investigation in a prospective trial in patients with high-risk of AF and AF complications. Larger sample size are needed to confirm these observations and ensure adequate statistical power, particularly in individuals with normal LAVI.

### Limitations

This study is not matched for important patient demographics such as gender-matching. It is plausible that LASI and LA-AT measurement might be influenced by the effect of atrial stunning. In our study we have no information regarding the exact timing of PAF episode in relation to date when the echocardiography was performed. Therefore, the observed associations in this study may reflect underlying cardiac characteristics associated with PAF rather than the burden of arrhythmia. It is essential to remark that no histological nor cardiac MRI validation for detection of LA fibrosis have been implemented in this study.

Several silent PAF patients were exclude due to missing/incomplete TTE examination influencing the feasibility of LASI and LA-AT and perhaps leading to selection bias and limit the generalizability of our findings. In addition, the overall feasibility of LASI and LA-AT was limited in both groups, which may further contribute to section bias. In subgroup analysis with normal LAVI, the feasibility of LASI and LA-AT presented < 23 individuals in the PAF group, as our power calculations demand $\:\ge\:$23 pariticpants to avoid type-2 error. Due to the sample size in our cohort with normal LAVI, our findings should be interpreted with caution and considered exploratory, despite differences of LA-AT and LASI between groups.

The enrichment criterion of the study regarding NT-proBNP levels may limit the generalizability of the findings to the broader elderly population. NT-proBNP levels are influenced by multiple clinical factors including BMI, serum albumin, long term blood glucose (HbA1c), renal function (eGFR) and anemia. In the present study, serum albumin, HBA1c and eGFR were not consistently available at the time of NT-proBNP sampling could therefore not be included as potential confounders. BMI was the only available parameters at the time of the TTE examination, whereas the diagnosis of anemia remained unchanged throughout the time of NT-proBNP sampling and TTE examination. Consequently, residual confounding related to clinical factors influencing NT-proBNP levels cannot be excluded.

Blood pressure and NT-proBNP levels were not collected at the time of TTE examinations, and there was a time discrepancy between the diagnosis of PAF and the TTE examination. NT-proBNP levels and blood pressure might differ at the registration of TTE examinations, however majority of patients were clinically stable, no HF diagnosis and LVEF > 50%. This temporal separation may limit the ability to relate echocardiographic findings to cardiac status at the time of AF detection. In addition, AF burden over time was not assessed. Although silent PAF was identified using a structured two-week screening protocol with intermittent ECG recordings at inclusion, this approach does not capture long-term AF burden and may lead to misclassification. However, medical record review in the PAF group including cardiology follow-up and ICD-10 coding, supported the perseverance of the PAF diagnosis at the time of the TTE examination. Finally, as this was a retrospective study, TTE examinations were not part of the original study design and were therefore not systematically aligned with other measurements which may have affected feasibility. The findings are therefore based on retrospective associations, which may limit casual interpretation and raise the possibility of reverse causality. Furthermore, the multivariate models included a limited set of clinical covariates, and residual confounding factors from unmeasured factors cannot be excluded. Although multicollinearity was low (VIF < 2), this does not eliminate the possibility of residual confounding.

## Supplementary Information

Below is the link to the electronic supplementary material.

Supplementary Material 1

Supplementary Material 2

Supplementary Material 3

## Data Availability

The data underlying this article cannot be shared publicly due to ethical/privacy reasons of individuals participated in the study. The data will be shared on reasonable request to the corresponding author.

## Abbreviations

AF: Atrial fibrillation
EF: Ejection fraction
GLS: Global longitudinal strain
HF: Heart failure
LASI: Left atrial stiffness index
LAVI: Left atrial volume index
LV: Left ventricle
MVC: Mitral valve closure
MVO: Mitral valve opening
NT-proBNP: N-terminal pro brain natriuretic peptide
PAF: Paroxysmal atrial fibrillation
PALS: Peak atrial longitudinal strain
LA-AT: Left atrial activation time
SV: Stroke volume
TDI: Tissue Doppler imaging
TTE: Transthoracic echocardiography
2D-ST: Two-dimensional speckle tracking
