# Supplementary material for: Echocardiographic markers for left atrial stiffness and left atrial conduction delay in patients with silent paroxysmal atrial fibrillation compared to age-matched controls
Source: Cardiovasc Ultrasound. 2026 Oct 28;24:32. doi: 10.1186/s12947-026-00395-1 (PMC13721264; doi:10.1186/s12947-026-00395-1)
Supplement: Supplementary file 1 [file 12947_2026_395_MOESM2_ESM.pdf]

Echocardiographic marker for left atrial stiffness and left atrial conduction delay in elderly patients with silent paroxysmal atrial fibrillation

STUDY POPULATION

General elderly population

Age: 76 ± 0.3 years
NT-proBNP: 125-900ng/L

ECHOCARDIOGRAPHIC MARKERS

LASI

Cutoff > 0.54

Sensitivity 75 %
Specificity 90 %
AUC 0.88

Marker of
LA mechanical dysfunction

LA-AT

Cutoff > 139 ms

Sensitivity 66 %
Specificity 88 %
AUC 0.81

Marker of
LA electrical remodeling

Despite normal LAVI (≤ 34 ml/m²)

Identification of silent paroxysmal atrial fibrillation

ECHOCARDIOGRAPHIC MEASUREMENTS

LA-AT (Activation Time)

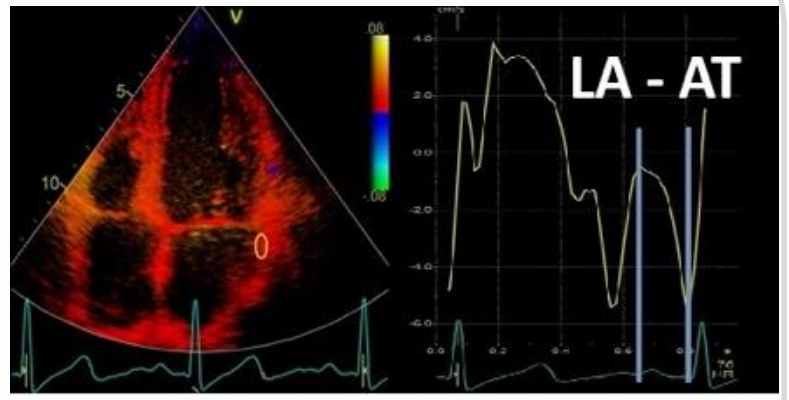

$$LASI = \frac{E/e'_{mean}}{PALS_{Biplane}}$$

E/e'_{mean} – lateral & septal
$PALS_{Biplane}$ – % of myocardial deformation
