# Supplementary material for: Echocardiographic markers for left atrial stiffness and left atrial conduction delay in patients with silent paroxysmal atrial fibrillation compared to age-matched controls
Source: Cardiovasc Ultrasound. 2026 Oct 28;24:32. doi: 10.1186/s12947-026-00395-1 (PMC13721264; doi:10.1186/s12947-026-00395-1)
Supplement: Supplementary file 2 [file 12947_2026_395_MOESM1_ESM.docx]

# **Lead author brief biography**

Larissa Bastos is a PhD. student in cardiovascular research in the department of Clinical Sciences at Karolinska Institutet Danderyd Hospital, Stockholm, Sweden. She has a BSc degree in Biomedical Science (2010) from Karolinska Institutet, Stockholm, Sweden, and a MSc degree in Cardiovascular Imaging (2018) from Örebro University, Sweden. She is currently working with Echocardiography and Cardiac MRI in the department of Clinical Physiology, Danderyd Hospital, Stockholm, Sweden. Her research interest includes the evaluation of atrial alterations with echocardiography in patients with atrial fibrillation, and studying the prediction of atrial fibrillation with quantitative echocardiographic parameters in the elderly population.
