# Supplementary material for: Echocardiographic markers for left atrial stiffness and left atrial conduction delay in patients with silent paroxysmal atrial fibrillation compared to age-matched controls
Source: Cardiovasc Ultrasound. 2026 Oct 28;24:32. doi: 10.1186/s12947-026-00395-1 (PMC13721264; doi:10.1186/s12947-026-00395-1)
Supplement: Supplementary file 3 [file 12947_2026_395_MOESM3_ESM.docx]

**Manus 2**

Graphical abstract text:

In a general elderly population without overt HF, increased left atrial stiffness index (LASI) and prolonged LA activation time (LA-AT) were associated with silent paroxysmal atrial fibrillation (PAF), despite normal LA volume index (LAVI). Together, these complementary markers may improve discrimination of silent PAF.
